# Comparative compositional analysis of cassava brown streak disease resistant 4046 cassava and its non-transgenic parental cultivar

**DOI:** 10.1080/21645698.2020.1836924

**Published:** 2020-11-04

**Authors:** H. Wagaba, P. Kuria, P. Wangari, J. Aleu, H. Obiero, G. Beyene, T. Alicai, A. Bua, W. Esuma, E. Nuwamanya, S. Gichuki, D. Miano, P. Raymond, A. Kiggundu, N. Taylor, B.M. Zawedde, C. Taracha, D.J. MacKenzie

**Affiliations:** a National Crops Resources Research Institute, Kampala, Uganda; b Kenya Agricultural and Livestock Research Organization, Nairobi, Kenya; c Institute for International Crop Improvement, Kakamega, Kenya; d Donald Danforth Plant Science Center, St. Louis, MO, USA; e Department of Plant Science and Crop Protection, University of Nairobi, Nairobi, Kenya; f AG SCI Consulting, LLC., Cottageville, SC, USA

**Keywords:** RNA interference (RNAi), genetically engineered, substantial equivalence, cassava

## Abstract

Compositional analysis is an important component of an integrated comparative approach to assessing the food and feed safety of new crops developed using biotechnology. As part of the safety assessment of cassava brown streak disease resistant 4046 cassava, a comprehensive assessment of proximates, minerals, amino acids, fatty acids, vitamins, anti-nutrients, and secondary metabolites was performed on leaf and storage root samples of 4046 cassava and its non-transgenic parental control, TME 204, collected from confined field trials in Kenya and Uganda over two successive cropping cycles. Among the 100 compositional components that were assessed in samples of 4046 and control TME 204 cassava roots (47 components) and leaves (53 components), there were no nutritionally relevant differences noted. Although there were statistically significant differences between the transgenic and control samples for some parameters, in most cases the magnitudes of these differences were small (<20%), and in every case where comparative literature data were available, the mean values for 4046 and control cassava samples were within the range of normal variation reported for the compositional component in question. Overall, no consistent patterns emerged to suggest that biologically meaningful adverse changes in the composition or nutritive value of the leaves or storage roots occurred as an unintended or unexpected consequence of the genetic modification resulting in 4046 cassava. The data presented here provide convincing evidence of the safety of 4046 cassava with respect to its biochemical composition for food and feed, and it could be considered as safe as its non-transgenic control.

## 1. Introduction

Cassava brown streak disease (CBSD) is a major constraint to cassava (*Manihot esculenta* Crantz) production in Sub-Saharan Africa, where the crop serves as a major staple food and an increasingly important source of industrial starch and ethanol.^1^ Foliar symptoms of CBSD can vary on different varieties, from non-apparent to interveinal chlorosis. However, the development of brown necrotic rot within the storage roots of affected plants causes the major impact of CBSD, rendering them inedible and without economic value. The causal viruses of CBSD, *Cassava brown streak virus* (CBSV) and *Ugandan cassava brown streak virus* (UCBSV) are transmitted by the whitefly vector *Bemisia tabaci* and the disease is also spread by farmers during their transport and planting of asymptomatic-infected stem cuttings used to establish next cropping cycle. Where highly susceptible varieties are grown, marketable yield losses due to CBSD can reach 100%.^2^ As of now, no CBSD-resistant cassava varieties are available for farmers.^3^

Under the Virus Resistant Cassava for Africa plus Iron and Zinc Enhancement (VIRCA Plus) project, RNA interference (RNAi) was employed to confer resistance to CBSD using an inverted repeat construct, p5001, containing fused coat protein (CP) encoding sequences from UCBSV and CBSV.^4^ Transgenic events of cultivar TME 204 expressing varying levels of small interfering RNAs (siRNAs) were established in multiple confined field trials (CFTs) at Namulonge, Uganda, and Mtwapa, Kenya, where they showed high levels of resistance to CBSD over several sequential cropping cycles compared to non-transgenic TME 204, which developed a high incidence of severe foliar and storage root symptoms.^5^ From these trials, event DPS-Ø4Ø46–8 (hereafter referred to as 4046 cassava) was selected for further characterization, including the generation of nutrient compositional data from samples collected over two consecutive cropping cycles at Kandara, Kenya, and Kasese, Uganda.

The safety assessment of genetically engineered (GE) foods includes a comparative assessment of the GE plant/food with its unmodified counterpart in order to arrive at a determination that the modified food is “as safe as” the conventional form. As established under international guidance issued by the Organization for International Cooperation and Development (OECD)^6^ and the Codex Alimentarius Commission,^7^ the safety assessment focuses on the defined differences between the GE food and its conventional counterpart. A compositional assessment of the edible portions of the GE plant in comparison to its unmodified counterpart is used to confirm the presence of intended changes, if any, and is part of the weight of evidence to evaluate whether there were any unintended, unexpected, consequences of the genetic modification. Analyses are conducted on key nutrients, anti-nutrients, and secondary metabolites as recommended in consensus documents on compositional considerations for new plant varieties published by the OECD, including cassava,^8^ with the aim of identifying whether there are any biologically meaningful differences between the new transgenic event and its conventional counterpart. Evaluating the biological relevance of any observed changes in a compositional component takes into account the range of natural variation for that parameter within the crop species as reported in the scientific literature and/or contained in public databases.^9^ The present study was performed to determine whether the storage roots and leaves derived from 4046 casava, which are commonly used as food or animal feed, were compositionally equivalent to these same materials produced from conventionally bred cassava.

## 2. Materials and Methods

### 2.1. Experimental Design – Field Phase

#### 2.1.1. Plot and Planting Information

The experimental CFTs to generate materials for compositional analysis were established over two cropping cycles, extending from October 2016 through September 2018 at Kandara, and from April 2017 through December 2018 at Kasese. The trials at Kandara and Kasese were conducted under the authorization and supervision of the Kenya National Biosafety Authority (NBA) and the Uganda National Biosafety Committee (NBC), respectively. Planting materials were acquired from selected stem cuttings generated from previous event selection CFTs at these sites. Woody stems were selected and segmented to generate uniform 25–30-cm long segments, each possessing a minimum of five nodes. Trials were planted with four replicates of event 4046 and TME 204 non-transgenic control cassava established in a randomized complete block design. Each plot of 25 plants was set up as five rows with 1 m × 1 m spacing for a total plot area of 16 m${\;}^{2}$. The second cycle planting was established using stem materials obtained from the first season. Prior to planting at Kandara, stem cuttings were treated with Marshal® 250EC insecticide (carbosulfan) (10 ml/16 L of water) and RIDOMIL GOLD® MZ 68 WG fungicide (40 g/16 L water) to protect against common soil pests and diseases. At Kasese, stems were directly planted without chemical pre-treatment. The internal nine plants of each plot of 25 plants were used for leaf and storage root sample collection for composition analysis.

#### 2.1.2. Maintenance of Field Plots

During the growing season, the field was kept free of weeds through routine weeding using a hand hoe and by hand pulling. The experimental area was visually inspected for the presence of pests and diseases immediately after sprouting, and every month thereafter. At Kasese, drip irrigation was used whenever necessary at a rate of 1.5 kg/m${\;}^{3}$ per hour at planting and three times a week for the first three months, depending on the weather. No fertilizer was applied at either location. Normal pest control and maintenance practices, consistent with good agricultural practice for cassava production, were used to produce the crop. As necessary, pest control products were applied to manage sucking and caterpillar pests, whiteflies, spider mites, thrips, leaf miners, and various mites. All maintenance practices were applied uniformly to the entire field area. The cropping cycle was approximately 12 months from planting to harvest.

#### 2.1.3. Sample Collection – Leaves

Samples of fully expanded leaves were obtained prior to root harvesting from each of nine plants (3 × 3 grouping) from the center of each plot of 25 plants and pooled to create a composite sample of at least 200 g fresh weight in a labeled plastic bag, which was immediately placed into a field cooler containing dry ice. Coolers were shipped by surface transport to the research laboratories located at the National Crops Resources Research Institute (NaCRRI) and the Kenya Agricultural & Livestock Research Organization (KALRO) for the Kasese and Kandara samples, respectively.

For cyanide analysis, leaves were harvested from three plants within the central nine plants in a plot, pooled together, and randomly mixed in a Ziploc plastic bag. From this composite sample, a 25 g sample was homogenized immediately in 25 ml of 0.1 M phosphoric acid pH 1.5 using a mortar and pestle. The homogenates were subsequently transferred into 50 ml Falcon tubes and sealed until further processing in the laboratory. For each plot, triplicate samples were taken.

#### 2.1.4. Sample Collection – Roots

On the day of harvest, 1–3 marketable storage roots with a minimum length of 16 cm and widths exceeding 3 cm were obtained from three plants from the central nine plants of each replicated plot. Roots were peeled and sliced transversely into segments of 1–1.5 cm thickness and combined into a bulk collection bag such that each bag contained a minimum of 600 g fresh weight of composited root tissue. Labeled sample bags were placed into field coolers containing dry ice and shipped by surface transport to NaCRRI and KALRO for the Kasese and Kandara samples, respectively.

For cyanide analysis, storage root samples were taken randomly from three plants of the internal nine plants in a plot. The roots were peeled using a kitchen knife, and washed with tap water. A 25-g composited sample was taken in triplicate and each was homogenized in 25 ml of 0.1 M phosphoric acid pH 1.5, transferred into a 50 ml Falcon tube and transported to the laboratory.

#### 2.1.5. Sample Handling and Processing

Upon receipt at the NaCRRI and KALRO laboratories, all leaf and root tissue samples were placed into cold storage at – 80°C until further processing. Except for the samples for cyanide analysis, frozen samples of leaf and root tissue were lyophilized and vacuum packaged and stored at room temperature until shipment to EPL Bio Analytical Services (Niantic, IL) for compositional analysis.

### 2.2. Compositional Parameters

The parameters chosen for compositional analysis of leaf and storage root samples (Table 1) were based on the OECD consensus document on compositional considerations for new cassava varieties^8^ and included proximates (moisture, protein, fat, fiber, and ash), starch, minerals, fatty acids, amino acids, vitamins, anti-nutrients, and toxicants.

**Table 1.** Compositional parameters analyzed in storage roots and leaves derived from event 4046 and control cassava

Tissue	Class	Analytes
roots, leaves	proximates	moisture, crude protein, crude fat, ash, acid detergent fiber (ADF), neutral detergent fiber (NDF), and crude fiber
roots	polysaccharides	starch
roots, leaves	minerals	calcium, phosphorus, magnesium, and iron
roots, leaves	fatty acids	caprylic (C8:0), capric (C10:0), lauric (C12:0), myristic (C14:0), pentadecanoic (C15:0), palmitic (C16:0), palmitoleic (C16:1 ∆9); heptadecanoic (C17:0), stearic (C18:0), oleic (C18:1 ∆9), linoleic (C18:2 ∆9,12), α-linolenic (C18:3 ∆9,12,15), arachidic (C20:0), eicosenoic (C20:1), eicosadienoic (C20:2 ∆11,14), eicosatrienoic (C20:3 ∆11,14,17), arachidonic (C20:4 ∆5,8,11,14), behenic (C22:0), erucic (C22:1 ∆13), lignoceric (C24:0), and nervonic (C24:1 ∆15)
roots, leaves	amino acids	lysine, arginine, glycine, histidine, isoleucine, leucine, phenylalanine, threonine, valine, alanine, aspartic acid, glutamic acid, proline, serine, tyrosine, cystine, methionine, and tryptophan
roots, leaves	vitamins	thiamine (B1), riboflavin (B2), niacin (B3), β-carotene, and vitamin C
leaves	anti-nutrients	soluble and insoluble tannins, and phytic acid
roots, leaves	toxicants	hydrogen cyanide (HCN)

### 2.3. Compositional Analyses

With the exception of hydrogen cyanide (HCN), all compositional analyses were performed by EPL Bio Analytical Services (Niantic, IL) using methods published by the Association of Official Analytical Chemists (AOAC), the American Oil Chemists’ Society (AOCS), or the American Association of Cereal Chemists (AACC).

#### 2.3.1. Proximate and Mineral Analyses

Samples of cassava leaves and roots were assayed to determine the percentage of moisture by gravimetric measurement of weight loss after drying in a forced air or vacuum oven, respectively ^10^. Ash determination in cassava root and leaf samples was by gravimetric measurement of the weight loss after ignition in a muffle furnace.^11^ The analytical procedure for crude protein determination in cassava root and leaf utilized an automated Kjeldahl technique based on a method provided by the manufacturer of the titrator unit (Foss-Tecator).^12^ To determine crude fat, samples were hydrolyzed with 3 N HCl at 90°C for 80 minutes, followed by extraction with a petroleum ether:ethyl ether:ethyl alcohol solution at 90°C for 60 minutes. After extraction, the samples were oven dried and the crude fat content was determined gravimetrically.^13,14^ The analytical procedure for crude fiber determination in cassava root and leaf was based on methods provided by the manufacturer of the extraction apparatus (Ankom Technology).^15^ Samples were analyzed to determine the percentage of neutral detergent fiber (NDF) by digesting with a neutral detergent solution, sodium sulfite and α-amylase. After rinsing with deionized water, the remaining residue was dried and weighed to determine the NDF content.^16,17^ For the determination of acid detergent fiber (ADF), samples were digested with an acid detergent solution, and after rinsing with deionized water, the remaining residue was dried and weighed.^18^ Mineral analyses were performed by inductively coupled plasma optical emission spectroscopy (ICP-OES) based on methods published by,^19^and CEM Corporation. ^20^

#### 2.3.2. Total Starch in Cassava Root

The analytical procedure for total starch determination in cassava root was based on a method provided by AOAC.^21^ Total starch is composed of resistant starch, which are carbohydrates that do not break down into sugar and are not absorbed by the small intestine, and nonresistant (digestible) starch. Nonresistant starch was extracted and converted to D-glucose by using pancreatic amylase and incubating in a shaking water bath overnight. Resistant starch was dissolved in 2 M KOH with vigorous stirring. The solution was neutralized with acetate buffer and the starch was quantitatively hydrolyzed to D-glucose with amyloglucosidase. The D-glucose in both nonresistant and resistant starch was determined with glucose oxidase/peroxidase reagent and the absorbance was measured on a spectrophotometer at 510 nm.

#### 2.3.3. Fatty Acids Determination in Cassava Root and Leaf

Following microwave-assisted ether extraction and saponification with 0.5 N NaOH in methanol, free fatty acids were converted to their fatty acid methyl ester derivatives and analyzed by GC-FID.^22,23^ Concentrations of each fatty acid were reported on a percent (%) of total basis.

#### 2.3.4. Amino Acids Determination in Cassava Root and Leaf

Along with tryptophan, cystine, and methionine, 15 additional amino acids were determined. The analytical procedure for analysis of these amino acids in cassava root and leaf was based on methods obtained from the Waters Corporation^24^ and described by.^25^ Cystine was converted to cysteic acid and methionine was converted to methionine sulfone. Following acid hydrolysis, free acids were converted to the 6-aminoquinolyl-N-hydroxysuccinimidyl carbamate derivatives, which were analyzed by reverse-phase ultra-high-performance liquid chromatography (UPLC) with UV detection. Tryptophan determination was based on an established lithium hydroxide hydrolysis procedure with reverse phase UPLC with UV detection.^26^

#### 2.3.5. Vitamins in Cassava Root and Leaf

For the determination of vitamins B1 and B2 (thiamine and riboflavin), cassava root and leaf samples were extracted with 10% acetic acid:4.3% trichloroacetic acid solution. A 50 – fold dilution was performed, and then the samples were analyzed by reverse-phase HPLC tandem mass spectrometry (MS/MS).^27^ Niacin (vitamin B3) was determined using a microbiological assay with *Lactobacillus plantarum* as described in.^28^ The determination of vitamin C was based on a method published by.^29^ The samples were extracted with 5% meta-phosphoric acid with tris(2-carboxyethyl)phosphine and analyzed by reverse-phase HPLC-MS/MS. Beta-carotene content was determined by HPLC analysis of samples extracted with a 40:60 acetone:hexane with tert-butylhydroquinone.^30,31^

#### 2.3.6. Anti-Nutrients in Cassava Leaf

The analytical procedure for the determination of tannins in cassava leaf was based on previously published methods.^32–37^ Soluble condensed tannins (SCT) were extracted from defatted cassava leaf with 5.26 mM sodium meta-bisulfite in 70:30 (v/v) acetone:deionized water. The insoluble condensed tannins (ICT) remained in the pellet after extraction. Both SCT and ICT were hydrolyzed with 95:5 (v/v) butanol:concentrated HCl in the presence of an iron catalyst. The hydrolyzates were analyzed by UV-VIS spectrophotometry and reported as procyanidin B2 equivalents. Samples were analyzed to determine the amount of phytic acid by extracting the phytic acid with dilute HCl and isolating it using an aminopropyl silica solid-phase extraction column.^38,39^ Once isolated and eluted, the phytic acid was analyzed for elemental phosphorus by ICP-OES.

#### 2.3.7. Total Cyanide Content in Cassava Root and Leaf

The total cyanogenic potential of plant tissue samples was determined by enzymatically converting cyanogenic compounds to hydrocyanic acid, which was quantified spectrophotometrically based on the König reaction with chloramine T and isonicotinic/1,3-dimethylbarbituric acid reagents.^40^

### 2.4. Statistical Analysis

All statistical analyses were conducted in *R*,^41^ including the “lmerTest”^42^ package. For a given compositional analyte, data across locations and seasons were analyzed using the following linear mixed model:

$$y_{ijkm}=\mu_{i}+l_{j}+s_{k}+r_{m(jk)}+(\mu l{)}_{ij}+(\mu s{)}_{ik}+(ls{)}_{jk}+(\mu ls{)}_{ijk}+\varepsilon_{ijkm}$$

where $\mu_{i}$ denotes the mean of the $i^{th}$ entry (fixed effect), $l_{j}$ denotes the effect of the $j^{th}$ location (fixed effect), $s_{k}$ denotes the effect of the $k^{th}$ year (fixed effect), $r_{m(jk)}$ denotes the effect of the $m^{th}$ block for the $j^{th}$ location and $k^{th}$ year (random effect), $(\mu l{)}_{ij}$ denotes the interaction between entries and locations, $(\mu s{)}_{ik}$ denotes the interaction between the entries and years, $(ls{)}_{jk}$ denotes the interaction between locations and years, $(\mu sl{)}_{ijk}$ denotes the interaction between entries, years, and locations, and $\varepsilon_{ijkm}$ is the residual error.

The “lmer” procedure from the “lmerTest” package was used to fit the linear mixed model and to generate estimates of variance components and p-values. For each compositional parameter, the estimated marginal (EM)-mean value across sites and growing seasons was estimated from the corresponding statistical model for 4046 cassava and the control TME 204 cassava using the “emmeans” package.^43^

#### 2.4.1. Statistical Comparisons and Interpretations

The first step in the evaluation was to test for differences in mean values between the transgenic and control entries. Where a statistically significant difference (p-value < 0.05) was identified in the combined-sites, multi-year analysis, further context for interpreting the possible biological significance of the difference was gathered through comparisons with the range of values for each analyte reported in the OECD consensus document on new cassava varieties^8^ and other literature sources.^44,45^ Analyte values for event 4046 cassava that fell within the literature range for that analyte were considered to be within the range of natural variability of conventional cassava, and thus not a safety or nutritional adequacy concern.

## 3. Results and Discussion

### 3.1. Proximates and Minerals

The composition of cassava depends on the specific tissue (storage roots or leaves) and on several other factors, including agro-ecological growing conditions, germplasm, and plant age. Cassava roots are energy-dense, with a large amount of starch ranging from 70% to 85%, dry basis (DB), of which *ca*. 83% is amylopectin and *ca*. 17% is amylose. The protein content of cassava roots is significantly lower than for cereal grains (typically < 3%), and storage roots are low in fiber (NDF <10%) making them highly digestible. The crude fat content in cassava roots typically ranges from 0.3% to 3% on a dry matter basis,^8^ which is relatively low compared to maize and sorghum.

Cassava leaves are a rich source of protein, minerals, and some vitamins. Crude protein typically ranges between 15% and 36% DB^8^ and crude fat content is higher than in the storage roots, ranging between 4% and 16%. Fresh leaves have a high fiber content (*ca*. 17% DB), and digestibility is low (70–80% in young leaves, decreasing to 67% in old leaves).^8^

In the multi-year, combined-sites analysis for proximates and minerals, the only statistically significant differences observed between 4046 and control cassava samples were for crude fiber in storage roots, and crude fat and NDF in leaves (Table 2). The decrease in mean crude fiber in 4046 cassava root samples compared to control TME 204 samples was relatively small (< 18%) and the range of values for 4046 cassava was within the combined literature range. Thus, the difference in crude fiber measured in 4046 cassava roots compared to corresponding control samples was not biologically meaningful. Similarly, the decreases in crude fat (−6.8%) and NDF (−6.2%) between samples of 4046 and control cassava leaves were not considered nutritionally relevant as the mean concentrations of both parameters in 4046 cassava leaves were within the combined literature range for these analytes.

**Table 2.** Proximate, starch, and mineral composition of storage root and leaf samples derived from event 4046 and control TME 204 cassava

Component	Mean^a^	Range	Mean	Range	*p*-value^b^	Lit. Range^c^
	*Storage Root Samples*					
Ash (%DB)^d^	1.87	(1.49–2.2)	1.89	(1.61–2.37)	0.827	0.5–8.6
Crude Fat (%DB)	0.65	(0.36–1.07)	0.52	(0.22–1.15)	0.130	0.2–3.2
Crude Protein (%DB)	1.46	(0.96–1.92)	1.81	(1.18–3.38)	0.062	1.4–4.7
Starch (%DB)	85.9	(79.1–91.3)	85.6	(81.6–91.1)	0.850	69.1–88.6
ADF^e^(%DB)	1.93	(0.47–2.65)	2.17	(0.78–3.58)	0.380	2.5–8.4
NDF (%DB)	2.93	(2.07–3.57)	3.19	(2.13–4.19)	0.232	4.1–12.0
Crude Fiber (%DB)	1.62	(1.06–2.04)	1.97	(1.5–2.8)	0.001	0.8–8.2
Ca^f^(mg/100 g DB)	66.4	(52–89.6)	62.9	(45.5–82.7)	0.202	19–330
Fe (mg/100 g DB)	0.78	(0.34–1.93)	1.01	(0.31–2.69)	0.121	0.3–23
Mg (mg/100 g DB)	84.0	(71–107)	83.6	(66.1–114)	0.911	50–240
P (mg/100 g DB)	88.5	(38.5–158)	88.8	(40.3–164)	0.961	6–320
	*Leaf Samples*					
Ash (%DB)	5.48	(4.37–6.65)	5.57	(4.37–6.80)	0.391	4.9–16.1
Crude Fat (%DB)	8.74	(7.07–9.62)	9.38	(7.83–11.6)	0.023	4.0–15.6
Crude Protein (%DB)	28.6	(26.5–30.7)	29.2	(28.2–30.9)	0.219	14.7–36.4
ADF (%DB)	35.4	(25–43)	36.3	(17.5–48.6)	0.684	18.1–36.4
NDF (%DB)	28.8	(21.8–40.7)	30.7	(20.4–41.5)	0.029	24.0–58.8
Crude Fiber (%DB)	11.8	(9.78–14.9)	11.9	(10.3–16)	0.860	4.8–26
Ca (mg/100 g DB)	774	(507–1180)	762	(535–1190)	0.666	34–1600
Fe (mg/100 g DB)	15.5	(8.6–25.4)	15.8	(8.2–26)	0.722	0.4–200
Mg (mg/100 g DB)	320	(213–498)	335	(220–506)	0.439	200–1030
P (mg/100 g DB)	321	(181–431)	315	(236–414)	0.494	27–620

^a^Values represent the EM means of three replicate samples collected from each location where event 4046 and control TME 204 cassava were grown between 2016 and 2018 (n = 12 for each entry). For each analyte, the lowest and highest individual values across locations and years are shown in parentheses.

^b^Statistical significance of differences due to plant genotype were assigned at *p < *0.05.

^c^The combined literature range was derived from the OECD consensus document on new cassava varieties,^8^, ^44^, and 45 as available.

^d^Percent dry basis (%DB).

^e^ADF = acid detergent fiber; NDF = neutral detergent fiber.

^f^Ca = calcium; Fe = iron; Mg = magnesium; P = phosphorus.

### 3.2. Amino Acids

Amino acid concentrations in 4046 and control cassava storage root samples were not significantly different across locations and growing seasons, with only two exceptions (Table 3). Across both locations and years, the concentrations of proline and valine were *ca*. 6 percent lower in 4046 cassava storage root samples than in control samples. However, these small changes were not consistently observed between trial sites (data not shown) and were not biologically relevant as cassava roots are not a nutritionally important source of dietary amino acids due to low protein content (0.7–2%), and the mean values of proline and valine for both 4046 and control TME 204 cassava samples were similar to the ranges reported in the literature.^8,44,45^

**Table 3.** Amino acid composition of storage root and leaf samples derived from event 4046 and Control TME 204 cassava

	Event 4046		Control TME 204			
Component	Mean^a^	Range	Mean	Range	*p*-value^b^	Lit. Range^c^
	*Storage Root Samples (mg/100 g DB)*					
Methionine	24.0	(20–27.4)	24.0	(19.1–28.2)	0.960	19–27
Cystine	32.4	(19.6–42.1)	35.1	(21.5–45)	0.366	23–69
Lysine	39.2	(28.6–51.6)	42.1	(28.9–56.5)	0.169	43–109
Tryptophan	34.1	(20.9–44.9)	28.6	(18.5–40.1)	0.096	19–47
Arginine	161	(60.6–280)	257	(88.9–710)	0.084	145–340
Isoleucine	41.1	(35.8–50.7)	43.5	(36.2–50.6)	0.056	31–67
Histidine	33.8	(29.7–42.3)	36.6	(27.7–53.7)	0.171	20–50
Valine	52.3	(45.1–65.7)	55.7	(46.1–66.1)	0.041	54–87
Leucine	64.0	(56–78.6)	67.6	(56.9–80)	0.058	55–97
Threonine	57.6	(49.1–67.5)	59.8	(46.7–68.7)	0.271	30–69
Phenylalanine	52.4	(46.8–61.5)	53.9	(44.9–62.1)	0.356	41–65
Glycine	54.0	(49.7–64.8)	56.0	(47.3–66.7)	0.284	38–69
Alanine	50.8	(44.3–61.8)	52.7	(43.4–68.2)	0.264	48–94
Aspartic acid	163	(71.8–233)	164	(82.2–247)	0.895	68–196
Glutamic acid	283	(221–339)	313	(249–426)	0.066	124–512
Proline	39.5	(36.3–48.6)	42.0	(35.6–49.1)	0.040	20–82
Serine	65.4	(56.3–78.3)	69.6	(54.5–78.9)	0.055	40–82
Tyrosine	*<*LOQ–36.6^d^		*<*LOQ–35.5		NA^e^	0–42
	*Leaf Samples (% DB)*					
Methionine	0.54	(0.46–0.60)	0.55	(0.50–0.60)	0.826	0.28–0.51
Cystine	0.44	(0.29–0.50)	0.46	(0.38–0.50)	0.359	0.18–0.36
Lysine	1.82	(1.61–1.98)	1.83	(1.54–1.97)	0.849	0.97–1.92
Tryptophan	0.49	(0.35–0.58)	0.44	(0.38–0.54)	0.043	0.24–0.51
Arginine	1.66	(1.45–2.08)	1.68	(1.49–2.19)	0.458	1.02–1.64
Isoleucine	1.34	(1.15–1.45)	1.36	(1.08–1.55)	0.702	0.97–1.71
Histidine	0.66	(0.59–0.72)	0.66	(0.55–0.78)	0.816	0.28–0.69
Valine	1.73	(1.53–1.87)	1.74	(1.45–2.0)	0.727	0.99–1.64
Leucine	2.51	(2.27–2.69)	2.53	(2.11–2.9)	0.754	1.20–2.72
Threonine	1.27	(1.12–1.38)	1.28	(0.99–1.48)	0.708	0.82–1.38
Phenylalanine	1.61	(1.41–1.79)	1.63	(1.33–1.94)	0.723	0.92–1.58
Glycine	1.52	(1.37–1.65)	1.53	(1.26–1.77)	0.794	1.12–1.76
Alanine	1.66	(1.5–1.75)	1.68	(1.5–1.87)	0.478	1.18–1.74
Aspartic acid	2.81	(2.46–3.07)	2.85	(2.16–3.22)	0.563	2.40–2.50
Glutamic acid	3.31	(2.79–3.57)	3.35	(2.27–3.74)	0.785	1.99–2.78
Proline	1.41	(1.29–1.5)	1.42	(1.25–1.62)	0.733	0.88–1.05
Serine	1.23	(1.02–1.39)	1.24	(0.81–1.42)	0.779	0.97–1.68
Tyrosine	0.90	(0.78–1.02)	0.93	(0.80–1.15)	0.171	0.69–1.18

^a^Values represent the EM means of three replicate samples collected from each location where event 4046 and control TME 204 cassava were grown between 2016 and 2018 (n = 12 for each entry). For each analyte, the lowest and highest individual values across locations and years are shown in parentheses.

^b^Statistical significance of differences due to plant genotype were assigned at *p < *0.05.

^c^The combined literature range was derived from the OECD consensus document on new cassava varieties,^8^, ^44^, and 45 as available.

^d^LOQ = Limit of quantification, which for tyrosine was 34 mg/100 g DB.

^e^NA = Not applicable. Statistical analysis was not possible as *>*80% of the analytical values were below the LOQ.

Although cassava roots do not have a well-balanced amino acid profile and are of lower nutritional value because of low protein quantity, cassava leaves have good protein content and are source of essential amino acids. The amino acid composition of 4046 and control TME 204 cassava was quite similar, except for tryptophan, which was elevated by approximately 11% in samples of 4046 cassava leaves (Table 3). This difference was not consistently observed between locations or growing seasons (data not shown), and was largely the result of a 27% higher tryptophan content measured in event 4046 leaves collected from the Kandara location in 2017. The mean values for tryptophan measured for both event 4046 and control cassava leaf samples were within the range of natural variation reported in the literature.^8,44,45^

### 3.3. Fatty Acids

The major fatty acids of cassava root meal lipid are oleic (C18:1 Δ9), linoleic (C18:2 Δ9,12), palmitic (C16:0), and α-linolenic (C18:3 Δ9,12,15) acids. Together, these four fatty acids typically comprise more than 90% of the total fatty acids in cassava storage root meal.^8^

The desaturation of oleic acid to form linoleic acid, and its subsequent desaturation to form linolenic acid, occurs only in plants; hence, both linoleic and α-linolenic acids are essential fatty acids for mammals. Other polyunsaturated and longer-chain polyunsaturated fatty acids can all be synthesized by mammals from dietary sources of α-linolenic and linoleic acid. Additionally, the synthesis of palmitoleic (C16:1 Δ9) and saturated fatty acids with chain lengths greater than 18 (e.g., C20:0, C22:0, C24:0) can be accomplished in mammals through *de novo* fatty acid synthesis without dietary requirements for palmitic and stearic acids, respectively. Hence, small changes in the concentrations of these non-essential fatty acids in samples from 4046 cassava roots or leaves relative to its parental control would have little or no biological significance to either humans or animals consuming 4046 cassava products.

Across years and locations, the only statistically significant differences noted between 4046 and control storage root samples were in the minor saturated fatty acids, stearic (C18:0) and behenic (C22:0), which were elevated by 4.3% and 3.9%, respectively, in 4046 cassava samples (Table 4). The same analysis of cassava leaf samples revealed statistical differences in the concentrations of three minor fatty acid constituents, myristic (C14:0), arachidonic (C20:4), and nervonic (C24:1), which together comprise less than 1.5% of the total fatty acids. None of these differences were considered nutritionally relevant.

**Table 4.** Fatty acid composition of storage root and leaf samples derived from event 4046 and control TME 204 cassava

	Event 4046		Control TME 204			
Component	Mean^a^	Range	Mean	Range	*p*-value^b^	Lit. Range^c^
	*Storage Root Samples (% total fatty acids)*					
Lauric (C12:0)	0.24	(0.14–0.51)	0.19	(0.13–0.23)	0.082	
Palmitic (C16:0)	24.4	(21.8–27.7)	24.2	(20.8–27.4)	0.225	12.3–31.0
Palmitoleic (C16:1)	0.21	(0.17–0.25)	0.20	(0.17–0.23)	0.118	4.1
Heptadecanoic (C17:0)	0.34	(0.26–0.44)	0.33	(0.26–0.44)	0.419	0.5
Stearic (C18:0)	2.90	(2.50–3.17)	2.78	(2.29–3.12)	0.005	2.7–6.0
Oleic (C18:1)	31.4	(21.8–42.6)	32.1	(22.3–38.6)	0.158	19.6–37.5
Linoleic (C18:2)	30.9	(23.3–36.6)	30.5	(24.8–37.2)	0.354	14.5–63.1
Linolenic (C18:3)	7.54	(3.34–10.5)	7.71	(4.46–10.2)	0.403	1.2–7.9
Arachidic (C20:0)	0.35	(0.28–0.40)	0.36	(0.30–0.42)	0.215	
Eicosenoic (C20:1)	0.46	(0.30–0.67)	0.46	(0.24–0.58)	0.736	
Behenic (C22:0)	0.80	(0.62–1.01)	0.77	(0.58–0.94)	0.006	
Erucic (C22:1)	0.10	(0.05–0.17)	0.12	(0.09–0.17)	0.199	
Lignoceric (C24:0)	0.32	(0.19–0.43)	0.32	(0.23–0.42)	0.640	
	*Leaf Samples (% total fatty acids)*					
Myristic (C14:0)	0.17	(0.14–0.23)	0.16	(0.12–0.24)	0.018	
Palmitic (C16:0)	14.8	(11.8–17.3)	14.5	(12.8–18.4)	0.243	
Palmitoleic (C16:1)	0.45	(0.28–0.61)	0.44	(0.34–0.59)	0.147	
Heptadecanoic (C17:0)	0.25	(0.18–0.34)	0.25	(0.19–0.43)	0.525	
Stearic (C18:0)	2.03	(1.59–2.44)	2.03	(1.64–2.71)	0.836	
Oleic (C18:1)	3.48	(2.83–4.45)	3.51	(2.43–4.96)	0.736	
Linoleic (C18:2)	13.7	(11.9–16.0)	13.1	(10.7–17.4)	0.113	
Linolenic (C18:3)	61.9	(55.1–68.1)	62.4	(50.6–67.9)	0.402	
Arachidic (C20:0)	0.35	(0.22–0.54)	0.35	(0.22–0.54)	0.850	
Eicosenoic (C20:1)	0.16	(0.12–0.23)	0.16	(0.11–0.26)	0.938	
Eicosadienoic (C20:2)	0.18	(0.14–0.21)	0.17	(0.14–0.23)	0.245	
Arachidonic (C20:4)	0.28	(0.25–0.30)	0.26	(0.22–0.30)	0.006	
Behenic (C22:0)	0.78	(0.60–1.04)	0.80	(0.61–1.1)	0.451	
Erucic (C22:1)	0.08	(0.05–0.10)	0.07	(0.05–0.09)	0.101	
Lignoceric (C24:0)	0.56	(0.50–0.63)	0.59	(0.47–0.87)	0.331	
Nervonic (C24:1)	0.77	(0.31–1.49)	1.21	(0.14–5.2)	0.030	

^a^Values represent the EM means of three replicate samples collected from each location where event 4046 and control TME 204 cassava was grown over two consecutive seasons between 2016 and 2018 (n = 12 for each entry). For each analyte, the lowest and highest individual values across locations and years are shown in parentheses. The concentrations of the following fatty acids were below the lower limit of quantification (LOQ) in storage root samples and are not reported: caprylic (C8:0); capric (C10:0); myristic (C14:0); pentadecanoic (C15:0); eicosadienoic (C20:2); eicosatrienoic (C20:3); arachidonic (C20:4); and nervonic (C24:1). In leaves, the concentrations of the following fatty acids were below the LOQ and are not reported: caprylic (C8:0); capric (C10:0); lauric (C12:0); pentadecanoic (C15:0); and eicosatrienoic (C20:3).

^b^Statistical significance of differences due to plant genotype were assigned at *p < *0.05.

^c^The combined literature range was derived from the OECD consensus document on new cassava varieties,^8^ and 57 as available.

### 3.4. Vitamins

Cassava roots contain relatively low levels of the B vitamins (thiamine, riboflavin, and niacin) and provitamin A (β-carotene), and a portion of these nutrients is lost during processing.^45^ The most abundant vitamin in cassava roots is vitamin C (ascorbic acid). The concentrations of β-carotene, thiamine, and niacin in storage root samples, and all vitamins tested in leaf samples, were not significantly different between 4046 and control cassava. The only significant difference was in vitamin C, which was elevated by 27.5% in samples of 4046 cassava roots (Table 5).

**Table 5.** Vitamins, anti-nutrients, and toxicants in storage root and leaf samples derived from event 4046 and control TME 204 cassava

	Event 4046		Control TME 204			
Component	Mean^a^	Range	Mean	Range	*p*-value^b^	Lit. Range^c^
*Vitamins* (mg/kg DB)	*Storage Root Samples*					
β-Carotene	0.25	(0.10–0.43)	0.26	(0.07–0.44)	0.505	0.024–0.198
Thiamine (vit. B1)	0.33	(0.20–0.56)	0.31	(0.19–0.68)	0.603	0.77–48
Niacin (vit. B3)	19.1	(13.1–24.8)	17.3	(11.2–29.7)	0.164	0.9–21.2
Ascorbic acid (vit. C)	871	(612–1388)	683	(389–1096)	0.003	50–511
*Toxicants* (mg/kg FWT)						
HCN^d^	94.9	(17.9–155.1)	128.8	(38.3–207.1)	0.040	4–500
*Vitamins* (mg/kg DB)	*Leaf Samples*					
β-Carotene	580	(509–729)	591	(469–818)	0.571	
Thiamine (vit. B1)	0.23	(*<*LOQ–0.28)	0.37	(*<*LOQ–0.53)	–^e^	
Riboflavin (vit. B2)	0.79	(0.49–1.69)	0.85	(0.46–1.52)	0.362	
Niacin (vit. B3)	74.0	(56.3–86.1)	79.8	(62.3–128)	0.232	
Ascorbic acid (vit. C)	14980	(9571–22240)	13570	(6152–18400)	0.090	
*Anti-Nutrients* (%DB)						
Tannins (soluble)	4.24	(2.61–5.64)	4.31	(2.33–6.31)	0.795	2.6–15.6
Tannins (insoluble)	1.61	(1.23–2.37)	2.32	(1.76–3.41)	0.003	0.1–3.8
Phytic acid	0.65	(0.53–0.80)	0.63	(0.49–0.76)	0.542	0.11–0.25
*Toxicants* (mg/kg FWT)						
HCN	143.4	(83.3–171.9)	192.2	(174.4–214.1)	0.002	12.3–500

^a^Values represent the EM means of three replicate samples collected from each location where event 4046 and control TME 204 cassava was grown over two consecutive seasons between 2016 and 2018 (n = 12 for each entry). For each analyte, the lowest and highest individual values across locations and years are shown in parentheses. In storage root samples, all values for riboflavin were below the LOQ of 0.18 mg/kg dry weight.

^b^Statistical significance of differences due to plant genotype were assigned at *p < *0.05.

^c^The combined literature range was derived from the OECD consensus document on new cassava varieties,^8^, ^44^, and 45 as available.

^d^Values represent the EM means of four replicate leaf and root samples collected from the Kasese site in 2017, and three replicate leaf and root samples collected from Kasese in 2018 (n = 7 for each entry). The lowest and highest individual values across growing seasons are shown in parentheses.

^e^Greater than 80% of the values for thiamine were below the LOQ and were not amendable to statistical analysis.

### 3.5. Anti-Nutrients and Toxicants

In addition to the toxicant, HCN, derived from the deglycosylation of linamarin, the OECD recommends testing for tannins and phytic acid as the significant anti-nutrients in cassava leaves but not in roots.^8^

Tannins are considered anti-nutrients because they can interfere with the absorption of iron and other minerals as well as form complexes with dietary proteins, rendering them indigestible. Tannin concentrations are negligible in roots,^8^ and in fresh leaves can range from 2.6% to 15.6% DB.^44^ Cassava leaves also contain complexes between tannins and proteins (i.e., insoluble tannins) at concentrations ranging between 0.1% and 3.8% DB.^44^

Phytic acid (inositol hexakisphosphate; phytate when in salt form) is the main storage form of phosphorus in plant tissues, which is not in a bioavailable form for monogastric animals that lack the digestive enzyme phytase. Phytic acid also has a strong binding affinity for nutritionally important minerals such as calcium, magnesium, iron, and zinc, thus reducing the absorption of these minerals. Other than binding with minerals, phytic acid also binds to proteins, reducing their digestibility and thus amino acid bioavailability. Phytic acids can also reduce free ion radical generation and thus peroxidation of membranes by complexing iron, and phytate may protect against colon cancer.^46^

The comparison of tannin and phytic acid concentrations in leaf samples derived from 4046 and control cassava showed no significant differences in concentrations of soluble tannins and phytic acid, but did find a significant reduction (−30.6%) in the level of insoluble tannins in 4046 samples compared to control samples when analyzed across locations and growing seasons (Table 5).

The principal toxicants present in cassava roots and leaves are two cyanogenic glycosides, linamarin and lotaustralin (methyl linamarin), which are produced in all cassava tissues except seed, typically in a ratio of approximately 20:1^40^. Linamarin is synthesized in leaves and transported to the roots where it is a source of nitrogen for protein synthesis.

The cyanogenic glycosides are a group of nitrile-containing plant secondary compounds that yield cyanide (cyanogenesis) following their enzymatic breakdown. Linamarin is stored in the vacuoles of leaf and root cells. Rupture of vacuoles as a consequence of tissue damage releases linamarin, which is hydrolyzed by a cell wall-associated β-glucosidase (linamarase) to yield the unstable nitrile intermediate, acetone cyanohydrin and glucose. Acetone cyanohydrin spontaneously decomposes to acetone and hydrogen cyanide (HCN) at pH >5.0 or temperatures higher than 35 C, or enzymatically via hydroxynitrile lyase.^47^

The cyanogen content of cassava foods can be reduced to safe levels by maceration, soaking, rinsing and baking. The various toxic effects resulting from the consumption of cassava containing residual cyanogens, including the cyanide metabolic pathway and toxicology for humans and animals, have been reviewed by.^48^

Levels of total cyanogen content in cassava roots depend on the cultivar and have been reported to range between 4 mg/kg and 500 mg/kg on a fresh weight basis,^8,45^ with significantly higher levels in leaves. Sweet varieties of cassava (low cyanide content) contain less than 50 mg/kg HCN on a fresh weight basis^48–50^ and can be processed by roasting, boiling or baking, whereas the bitter varieties (high cyanide content) require more extensive processing. Effective measures for cassava root detoxification are a combination of peeling, soaking, macerating, drying, and boiling to ensure safe consumption. ^51,52^

In comparing samples derived from 4046 and control TME 204 cassava, the mean concentrations of HCN in both leaves and storage roots were significantly reduced by approximately 25–26% in 4046 samples (Table 5). The range of measured HCN concentrations in leaf and root tissue samples for both 4046 and control cassava was within the ranges of normal variation reported in the literature.

### 3.6. Conclusions

For new varieties without purposefully altered nutritional properties, such as those with introduced traits conferring disease and pest resistance and/or herbicide-tolerance, which make up the vast majority of currently cultivated genetically engineered crops, experience has shown that the incorporation of these traits has not meaningfully impacted either nutrient composition or the nutritive quality of the crop.^53,54^ As with products of conventional plant breeding, most compositional variation for genetically engineered crops is due to environmental and agronomic factors, and the parental germplasm.^55,56^

Among the 100 compositional components that were assessed in samples of 4046 and control TME 204 cassava roots (47 components) and leaves (53 components), there were only 13 statistically significant differences noted across locations and growing seasons. In most cases, the differences were relatively small, less than 20%, and in every case where significant differences were noted and comparative literature data were available, the mean values for 4046 and control cassava samples were within the range of normal variation reported for the compositional component in question. No consistent patterns emerged to suggest that biologically meaningful adverse changes in the composition or nutritive value of the storage roots or leaves had occurred as an unintended, unexpected, consequence of the genetic modification resulting in 4046 cassava.

The data reported here provide important evidence supporting the safety of new cassava varieties containing event 4046 as food, livestock feed, or in food processing. The introduction of CBSD-resistant cassava varieties will address one of the most important constraints to cassava production in East Africa and will significantly improve smallholder incomes and well-being.
